# Fiber-Based Triboelectric Nanogenerator for Mechanical Energy Harvesting and Its Application to a Human–Machine Interface

**DOI:** 10.3390/s22249632

**Published:** 2022-12-08

**Authors:** Chen-Kuei Chung, You-Jun Huang, Tun-Kai Wang, Yu-Lung Lo

**Affiliations:** Department of Mechanical Engineering, National Cheng Kung University, Tainan 701, Taiwan

**Keywords:** mechanical energy harvester, triboelectric nanogenerators, TENG, fiber, polyester, polydimethylsiloxane, PDMS, sensor, human–machine interface, HMI

## Abstract

Mechanical energy harvesters including piezoelectric nanogenerators, electromagnetic generators and triboelectric nanogenerators (**TENG**) used to convert the mechanical motion into electricity are more and more important in the recent decades. Specifically, the fiber-based TENG (**FTENG**) has gained considerable favors due to its flexibility, light weight, and high environmental tolerance for the wearable devices. The traditional FTENGs made of Teflon result in better performance but are not suitable for long-term wear in person. Here, we propose a novel FTENG using a flexible micro-needle-structured polydimethylsiloxane (**MN-PDMS**) together with the comfortable commercially available **2D-polyester fibers**, and **electroless nickel-plated cotton cloth** of which two are widely used in human daily life. The MN-PDMS is formed by a laser engraved mold for improving its output performance of FTENG compared to the flat-PDMS. The open-circuit voltage (Voc) and the short-circuit current (Isc) of MN-FTENG increased to 73.6 V and 36 μA, respectively, which are 34% and 37% higher than the flat-FTENG. In terms of power, the performance of MN-FTENG reaches 1.296 mW which is 89% higher than that of flat-TENG and it can also light up 90 LEDs. For application, human motion at the joints can be detected and collected with various signals that are used for the human–machine interface (HMI) through the cooperation of components for the Internet of Things (IoT). It can light up the LED bulb through MN-FTENG to potentially develop IoT HMI systems for human motion control of robot in the future.

## 1. Introduction

Mechanical energy harvesters [1,2] can convert mechanical motion into electricity during movement. The application of mechanical energy harvesters can be used for wearable self-powered sensing devices and to act as generators, which makes them have great potential as battery-free HMIs in the IoT. Typical mechanical energy harvesters including piezoelectric nanogenerators (PENG) [3,4], electromagnetic generators (EMG) [5,6]. Among them, the Triboelectric Nano-generator (TENG) [7] is a self-powered sensor [8,9] with high mechanical sensitivity and power generation efficiency. TENG has four different modes of power generation mechanisms [10], these are: vertical contact-separation mode, lateral sliding mode, single-electrode mode, and freestanding triboelectric-layer mode. These four modes of TENGs can almost correspond to all types of motion in human daily life. Compared with other mechanical energy harvesters, which often require the use of metal materials or ceramic materials, TENG can be composed of a variety of materials, including a variety of ductile materials. This makes TENG useful for wearable devices through material selection and creates a lot of convenience for a human’s daily life in the future.

The rise of flexible devices in recent years makes a new revolution in human–machine interfaces (HMIs) and internet of things (IoT) applications, which can meet people’s growing pursuit of a more convenient life [11,12,13]. However, traditional bulky and rigid electronic devices obstruct the compliant interfacing with human skin [14,15]. Therefore, conventional devices affect the comfort level of users to a large extent. To overcome these drawbacks, it is essential to develop flexible sensors with excellent mechanical flexibility and stretchability. These sensors attached to curved and dynamic surfaces, such as human skin, can continuously monitor physiological and environmental indicators in real time [16,17]. TENGs composed of flexible and stretchable materials have the potential to be used as sensors in wearable devices.

Among many studies [18,19], the fiber-based TENG (FTENG) has gained considerable favors among many teams in recent years due to its flexibility [20], light weight [21], high force sensitivity, and high environmental tolerance for inserting into clothing, insoles and other products as a wearable device and integrate into human beings without any sense of violation. FTENG can also be divided into 1D [22], 2D [23], and 3D [24] dimensions according to different weaving methods. This makes FTENG more suitable to use to collect mechanical energy and signals of human motion than other types of mechanical energy harvesters. Higher woven dimension provides better flexibility, stretchability, and effective contact area, but meanwhile, it also increases the process complexity and the cost of triboelectric layers. In our daily life, FTENG can be used as an HMI to operate mechanical operations. FTENG may collect mechanical energy during sensing the motion force and be used as a power supply system for wearable devices or as a human health monitoring system [25], and for the purpose of sensing or self-powering, better TENG output performance is necessary, and it is also necessary that it can only be used in practical applications if it meets the characteristics of durability and washability. However, in most of the wearable TENG studies, FTENG is assembled into TENG by using Teflon [26,27] or other materials that are not flexible and stretchable to be drawn and woven into textile-like triboelectric layers using various weaving techniques. This type of FTENG is not only complicated to manufacture, but also lacks high enough flexibility and stretchability. Therefore, this kind of FTENG is not suitable for a wearable device to be worn close to the body for a long time.

**Here**, we propose a novel FTENG using a flexible micro-needle-structured polydimethylsiloxane (MN-PDMS) together with the comfortable commercially available 2D-polyester fibers. We uniformly coat the flexible and extensible polydimethylsiloxane (PDMS) on the polyester fibers (PET) as the main material of FTENG. Additionally, an electroless nickel-plated cotton cloth is used as the conductive fiber here for the electrode which is widely used in human daily life. These ductile materials can greatly improve the flexibility and stretchability of FTENG. It is noted that the MN-PDMS may enhance the contact surface and energy harvesting performance of Al-PDMS TENGs [28,29] and is formed by a laser-engraved mold for improving its output performance of TENG compared to the flat-PDMS [30,31]. The FTENG with MN-PDMS here is named **MN-FTENG**. The MN-FTENG can be used for high-sensitivity motion tests such as human joint moment and applied on HMI with an Arduino controller. The open-circuit voltage (Voc) and the short-circuit current (Isc) of MN-FTENG increase to 73.6 V and 36 μA, which are 34% and 37% higher than the original FTENG because of the increase in effective surface area. Additionally, the output power of MN-FTENG reaches 1.296 mW with 89% higher than FTENG without microstructure. In addition, MN-FTENG can also light up 90 LEDs, it is 1.3 times of flat-FTENG. The MN-FTENG is used for detecting the human motion at joints to collect various signals those are used for the human–machine interface (HMI) through the cooperation of components for the potential Internet of Things (IoT). The LED bulb is lit up and controlled through MN-FTENG which implies the potential to develop HMI systems for the human motion control of robots in the future.

## 2. Experimental Procedures

### 2.1. The Material Selection and Assembly of MN-FTENG

Polyester fiber is a kind of linear polymer obtained by polycondensation reaction of saturated dibasic acid and dihydric alcohol. It is associated with an ester group “-COO-”, so it is commonly called polyester. Polyester fiber has high strength and elastic recovery ability, so it is flexible and stretchable. Unlike most of the studies, where the fiber material mostly acts as a shape-maintaining material, the fiber base in this study is also part of the triboelectric layer. Therefore, the effect of fiber materials on the mechanical energy harvesting performance must also be considered here. According to the Triboelectric Series [10], PET is the material which gains electrons easily and stays in the negative zone. When we use it as a triboelectric layer to make a wearable device and work with the human skin that easily lose electrons, it can produce a larger surface electrical difference than other cheap and common fiber material such as Nylon and cotton. This is helpful for greater mechanical energy harvesting. The polyester fiber used in this experiment is a kind of common commercially available twill weave polyester fiber. Its anti-wrinkle and high-strength properties can stabilize the film thickness in the subsequent coating process and improve the tensile resistance and dimensional stability of the triboelectric layer. The electrode used in this experiment is a commercially available conductive cloth, which is the composition of the metal nickel and the fiber by electroless plating. It is a flexible fiber material with conductivity similar to a metal material. By forming a metal film on each fiber, the characteristics of the metal can be imparted under the characteristics of the fiber. The composite process of attaching metal thin films to fibers by electroless plating can achieve the same conductivity as metal foils while maintaining flexibility and stretchability. The polymethylmethacrylate (PMMA) used in this experiment is the polymer with the best optical properties, with more than 90% light transmittance for light wavelengths from 340 nm to 1100 nm. PMMA can directly absorb a laser of specified wavelength to achieve a good etching effect. In this experiment, PMMA was processed by a laser-engraving machine as a mold, and PDMS was transferred over the mold. Since there is no mutual pollution and no serious loss, the PMMA mold can be reused several times. The polydimethylsioxane (PDMS) is a silicone elastomer material with a hydrophobic surface and a contact angle of about 100–120°. The polymer is thermally and chemically stable due to its Si-O bond strength and is easy to prepare. The PDMS used in this study is for the production of thin film rapid prototyping of TENG by mixing elastic agent and curing agent in a ratio of 10:1 by weight.

**Figure 1:**
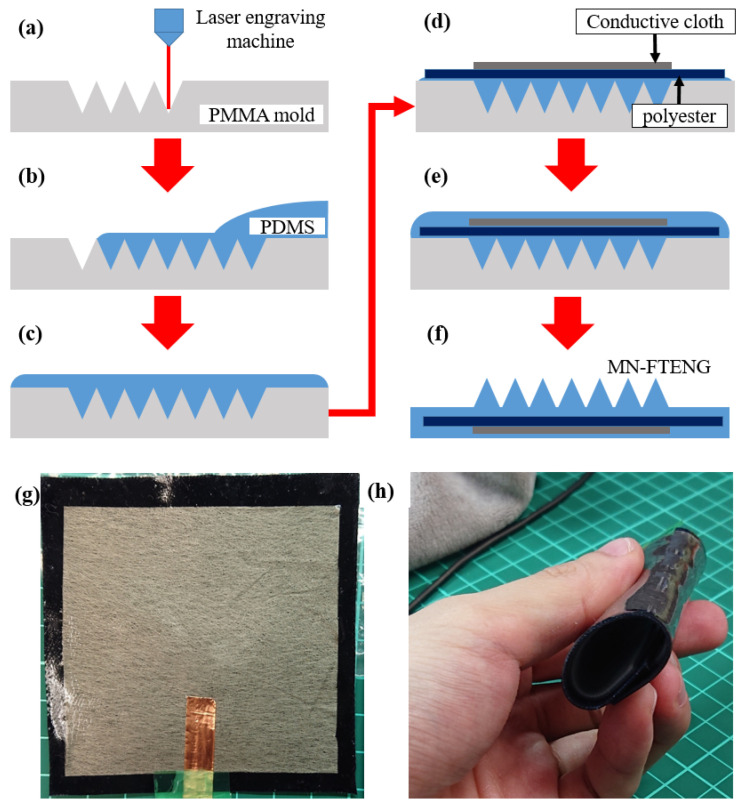
The process of preparing MN-FTENG and the appearance of MN-FTENG in this experiment: (**a**) Microstructures were fabricated on the PMMA mold with a laser engraving machine, (**b**) PDMS was poured into PMMA mold, (**c**) air bubbles was evacuated from PDMS in a vacuum chamber, (**d**) Lay polyester and conductive cloth on PDMS, (**e**) let PDMS fully penetrate into fiber in the vacuum chamber, (**f**) after baking and demolding, MN-FTENG is formed, (**g**) the appearance of MN-FTENG and (**h**) MN-FTENG can still be used after bending 360 degrees.

Figure 1a–f shows the manufacturing steps and process of MN-FTENG. First, the PMMA mold was processed by a laser engraving machine and ablated to form continuous microstructure grooves (Figure 1a). The PDMS and curing agent were mixed well and poured onto the PMMA mold (Figure 1b) and degassed for 10 min to fill the holes (Figure 1c). Then, the PET (90 × 90 mm) and conductive cloth (70 × 70 mm) were laid flat on the mold (Figure 1d) and kept some time for the PDMS stuck to the fiber structure (Figure 1e) and followed by putting it into an 80 °C oven for curing. After curing, the polyester fiber triboelectric generator with PDMS microneedle structure can be easily peeled from the mold and cut into 80 × 80 mm size to remove irregular burrs (Figure 1f). The completed MN-FTENG is shown in Figure 1g. With the stretchability and flexibility of the materials used, the device can be freely bent and recovered and can be used in a bent state, as shown in Figure 1h).

### 2.2. The Experiment and Measurement of MN-FTENG

**Figure 2:**
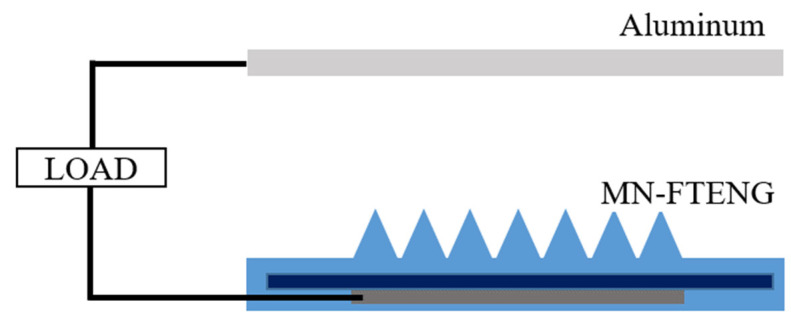
The electrical system of MN-FTENG. The two triboelectric layers of MN-FTENG are aluminum and MN-PDMS. Additionally, the two electrodes of MN-FTENG are aluminum and the conductive fiber inside the MN-FTENG. Since human skin has the positive triboelectric series, in the wearable device, the aluminum is replaced by human skin which becomes the other friction layer of MN-FTENG and turns the MN-FTENG into Single-electrode mode.

In order to simulate the motion form of TENG touching and separating with the skin, we use a TENG pneumatic cylinder test platform to achieve vertical contact-separation mode. The TENG pneumatic cylinder test platform uses the pneumatic cylinder (FESTO D:S-PAZ-DW20-100PPV, Esslingen, Germany). Figure 2 shows the schematic electrical system of MN-FTENG performance test. The two triboelectric layers of MN-FTENG, i.e., aluminum and MN-PDMS are stickled to the drive shaft and the stage, respectively. The two electrodes of MN-FTENG are aluminum and the conductive fiber inside the MN-FTENG. The test platform is actuated by air pressure (the input air pressure is 2 kg/cm^2^), and carried out under the conditions of 8 Hz, 16 N, and the distance between the test piece and the low-purity aluminum electrode is 25 mm. During the test, the aluminum electrode and the test piece are connected to the transient waveform recorder with copper wires to measure the open-circuit voltage signal and short-circuit current needs to be measured in parallel with a high resistance load. In addition, various DC performances of flat-FTENG and MN-FTENG can also be tested by simply connecting capacitors and Light Emitting Diodes (LEDs) through rectifying flat-FTENG or MN-FTENG’s signals by a bridge rectifier. Due to the high stability of this platform, the platform can also maintain performance without degradation during high cycle beating experiments to test the stability of MN-FTENG.

For the actual testing the performance of MN-FTENG as a wearable device in the HMI system, the aluminum electrode is replaced by human skin which becomes the other friction layer of MN-FTENG since human skin has the positive triboelectric series. Additionally, this turns the MN-FTENG into Single-electrode mode. Additionally, the MN-FTENG is cut into 30 mm × 80 mm for better wearability. The force sensitivity of MN-FTENG is confirmed through a dropping test of the weight drop from the fixed height of 3, 5, 7 and 10 cm. In this way, the MN-FTENG and the load cell will be impacted by the same force, and this force will be measured out through the load cell. The measured force ranges from approximately 0.5 to 10 N. The characterization of the microstructure of MN-FTENG was recorded by optical microscopy (OM, Olympus BX 51 M, Tokyo, Japan). The open-circuit voltage (Voc) and the short-circuit current (Isc) of MN-FTENG were recorded by an oscilloscope (HIOKI Memory HiCorder MR8870-20, Nagano, Japan). The electric output power of flat-FTENG and MN-FTENG can be calculated by the output voltage and output current under load by W = IV. Additionally, the development boards in HMI-related experiments are Arduino UNO.

## 3. Results and Discussions

### 3.1. The Output Performance of FTENG and MN-FTENG

**Figure 3:**
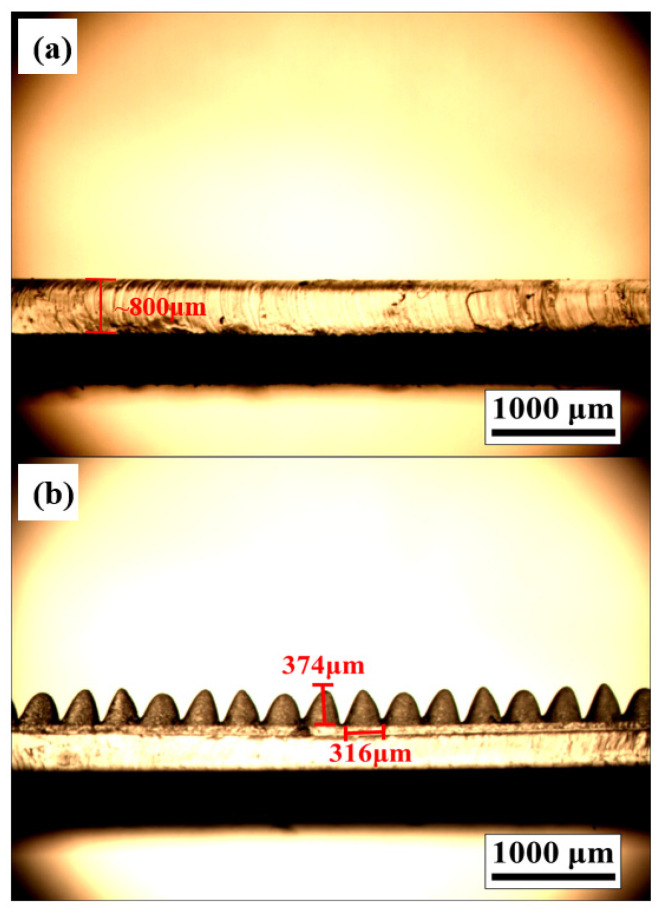
(**a**,**b**) OM diagram of smooth surface and 374 μm microneedle structure on PDMS. Both of these two samples are ~800 μm thick.

**Figure 4:**
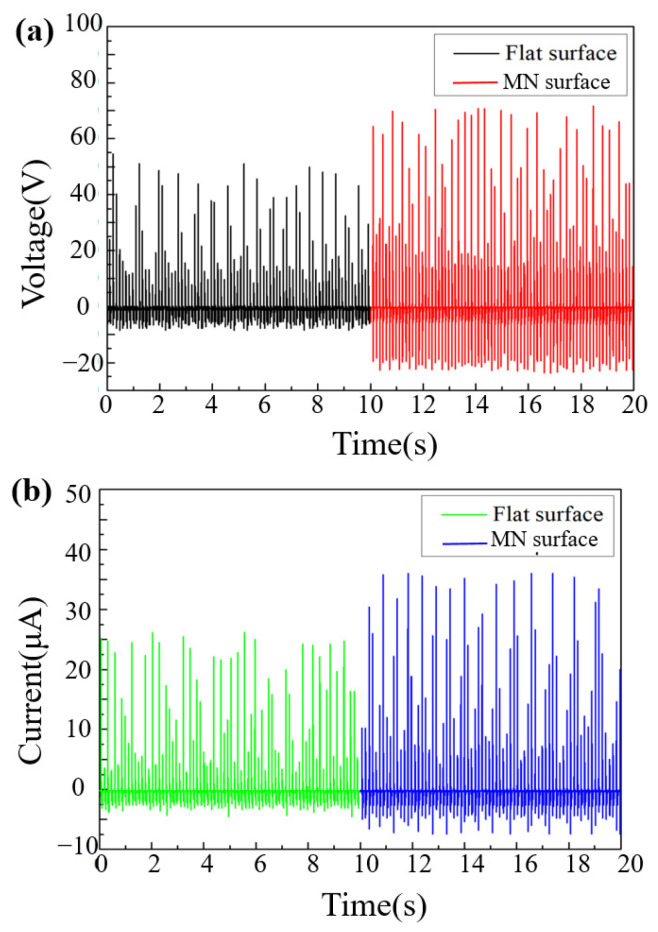
(**a**) Open circuit voltage and (**b**) current diagram of flat surface FTENG and 374 μm microneedled structure MN-FTENG.

The microstructures fabricated in this study are regulated by laser parameters of power, speed and density. The height and width of the microneedles of the PDMS mold after processing were measured by OM. As shown in Figure 3a,b, the surface of the flat and unstructured test piece is flat, and the microneedle test piece can see that its structure height is 374 μm. Both of their PDMS layers are about 800 μm thick and the whole triboelectric layers are about 4 mm thick. At the same time, by increasing the number of microneedles per unit area, a structure adjacent to the microneedles is fabricated to increase the contact area. The performance difference is compared between the microneedle fiber triboelectric nanogenerator (MN-FTENG) and the unstructured planar fiber triboelectric nanogenerator (flat-FTENG) in its open circuit voltage and short circuit current. From the open-circuit voltage and short-circuit current of Figure 4a,b, it shows that the voltage and current between FTENG and MN-FTENG have a clear trend of increasing. Compared with the flat-FTENG, the open circuit voltage of MN-FTENG increased from 54.6 V to 73.6 V. While the short-circuit current increased from 26.16 μA to 36 μA. It means that the MN-PDMS brings 34% higher open-circuit voltage signal and 37% higher short circuit signal to the MN-FTENG. From the above data, we can find that under the same mechanical force, the microstructure array can indeed increase the power generation performance. In addition, in the calculation of the current density, the MN-FTENG reaches a maximum of 7.34 mA/m^2^. As listed in Table 1, MN-FTENG with the microstructure can achieve better performance than some other FTENGs. In addition, FTENG without the microstructure also has a good enough performance in the field of fiber-based TENG. The higher output performance of MN-FTENG is attributed to the enhanced contact area and high triboelectric polarity difference between the MN-PDMS and Al tribo-layers, as well that both PDMS and PET are highly flexible to make more electron transfer and conduction during continuous contact-and-separate deformation compared to some materials.

**Figure 5:**
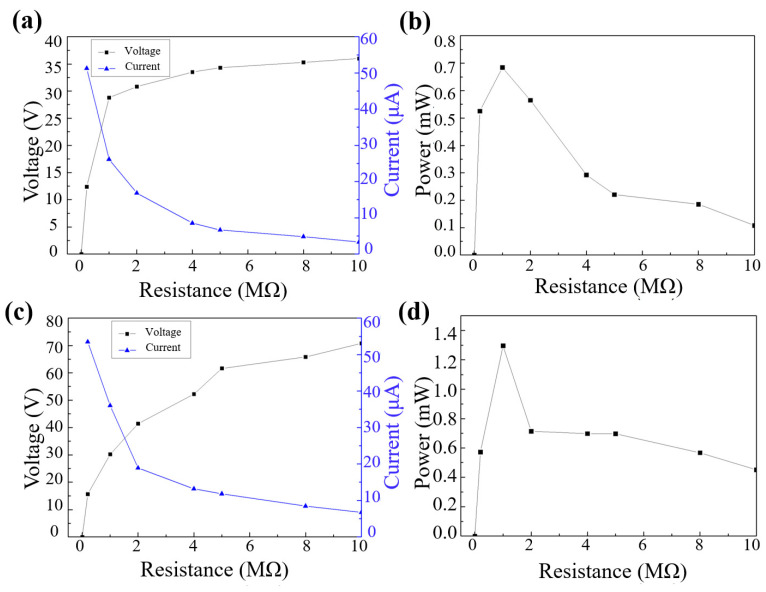
(**a**) Voltage–, current–resistance curve and (**b**) power–resistance curve of unstructured FTENG; (**c**) voltage–, current–resistance curve and (**d**) power–resistance curve of MN-FTENG. These four pictures show that the maximum power of MN-FTENG and FTENG appear at 1 MΩ, which are 0.68 mW and 1.296 mW.

As shown in Figure 5a,c, the voltage increases with the increase in the resistance until it is close to the open-circuit voltage; the current decreases with the increase in the resistance due to the ohmic loss. In the high resistance region, the current reduction becomes slow. After calculation, the maximum power of unstructured FTENG also appears at 1MΩ, which is 0.68 mW. Additionally, the power density is 0.139 W/m^2^ in Figure 5b. Additionally, Figure 5d shows the maximum power of MN-FTENG appears at 1 MΩ, which is 1.296 mW. Additionally, the power density is 0.264 W/m^2^. It can be seen that the microstructure increases the effective friction area, which can allow more PDMS area to rub against the Al electrode to generate more triboelectric charges, increase the charge density, increase the voltage and current, and finally increase the power by 189% and power density to 0.264 W/m^2.^ Higher output power enables MN-FTENG to drive more electrical appliances, which can reduce battery consumption and energy waste when applied in wearable HMIs in the future.

### 3.2. Durability and Washing Test

**Figure 6:**
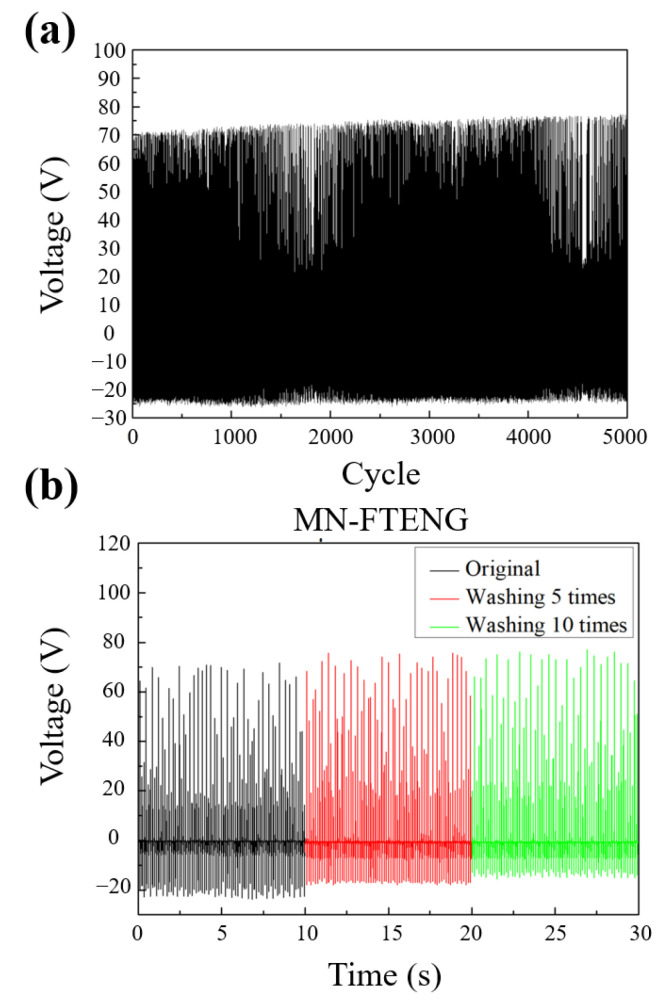
(**a**) The electrical performance of MN-FTENG is stable after 5000 cycles of beating test, and the inset is the enlarged waveform; (**b**) MN-FTENG has quite stable power generation performance in 0 times, 5 times and 10 times of cleaning tests.

TENG is essentially a green energy technology that converts mechanical energy into electrical energy, so its structural durability and output stability under repeated mechanical force operation will be one of its performance indicators. Therefore, in this section, MN-FTENG will be tested for long-term durability and the stability of its output performance will be observed. Continuous recording of its output voltage waveform in 5000 cycles of beating test, it can be seen that MN-FTENG has quite stable output performance, and there is no phenomenon of output performance degradation due to structural damage, as shown in Figure 6a. This indicates that the MN-FTENG proposed in this study has excellent structural stability in continuous long-term durability tests and has the advantage of stable output in practical use, which can be used as a self-powered system or sensor application.

The fiber-based TENG has the characteristic of flexibility and is often used in human body wear in many applications. However, if it is to be practically applied to general clothing, its performance stability after cleaning will be a key indicator whether it has the feasibility of physical application. Therefore, this section will discuss whether the power generation efficiency of MN-FTENG declines after cleaning to check its stability. In the cleaning process of this experiment, first rinse the test piece with clean water and rub the surface with detergent, put it into a beaker containing a detergent aqueous solution and ultrasonically shake it for 5 min, then rinse the excess foam with clean water, blow off excess water with an air gun. Finally, the test piece is dried at 55 °C for 10 min to remove residual moisture. The voltage data of the test piece cleaned 0 times, cleaned 5 times, and cleaned 10 times were used as the index to observe the performance difference. From Figure 6b, it can be seen that the voltage output of MN-FTENG is quite stable after 5 and 10 times of cleaning, and the voltage difference is within ±5 V, which shows the cleaning stability of MN-FTENG and its potential of daily wearable sensors which must be easy to clean as the clothing is frequently worn by humans and soiled.

### 3.3. Energy Storage Characteristics and LED Lighting

**Figure 7:**
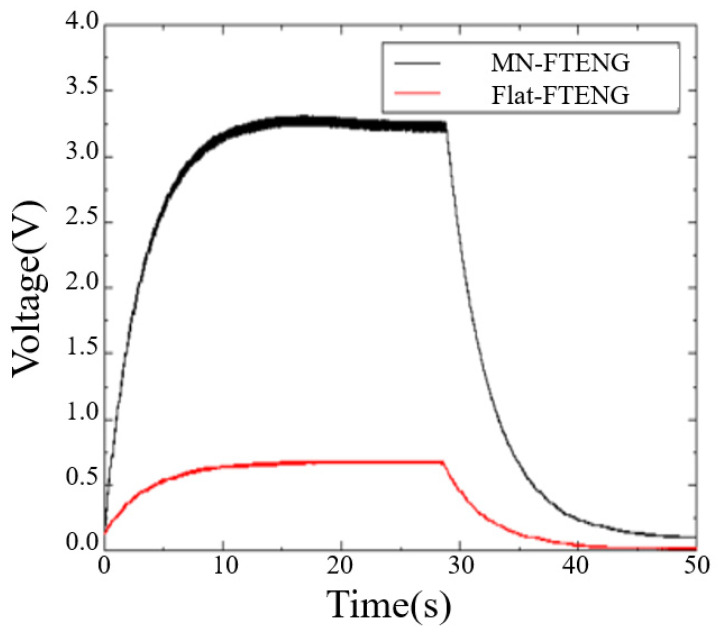
Charge–discharge curves of MN-FTENG and FTENG with 3.3 μF capacitors, it shows that the unstructured FTENG can charge 3.3 μF to 0.7 V in 20 s, while the 374 μm MN-FTENG can charge 3.3 μF to 3.3 V in 15 s. Additionally, the unstructured FTENG takes 14 s from saturation to full discharge, and the MN-FTENG takes more than 21 **s** from saturation to full discharge.

To supply the output voltage of TENG to small electronic devices, TENG can be used to charge and stabilize the capacitor and then drive the small components with a stable DC voltage. Therefore, studying the efficiency of MN-FTENG in capacitor charging and discharging is also an indicator for confirming the practical applicability of MN-FTENG. As shown in Figure 7, the unstructured FTENG can charge 3.3 μF to 0.7 V in 20 s, while the 374 μm MN-FTENG can charge 3.3 μF to 3.3 V in 15 s. A higher capacitor charging voltage can make the mechanical energy harvester drive more electronic components while sensing, as shown in Figure 7. While the unstructured FTENG takes 14 s from saturation to full discharge, and the MN-FTENG takes more than 21 s from saturation to full discharge. A longer discharge time can make the electronic components have a longer operating time.

**Figure 8:**
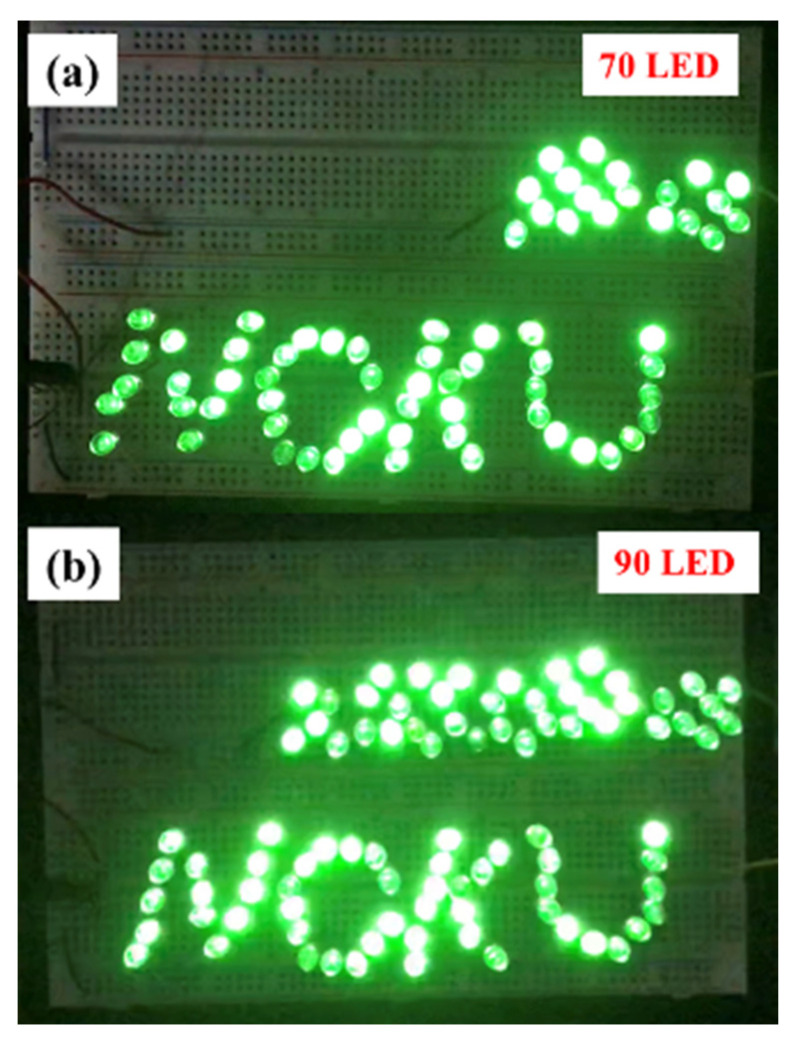
(**a**) Unstructured FTENG can drive 70 LEDs and (**b**) MN-FTENG can drive 90 LEDs.

The MN-FTENG fabricated in this study can be used to drive LEDs as lighting or indicator lights, and the Voc and Isc of the TENG will directly affect the number and brightness of LEDs. In this experiment, the green LEDs are connected in series, and the TENG is rectified by a bridge rectifier and then directly output to drive the LEDs. The contribution of microstructures to the output performance can be directly judged through the number of emitting LEDs. It can be seen in Figure 8 that the MN-FTENG can light up 90 LEDs, while the unstructured FTENG can light up 70 LEDs. Therefore, it can be judged that the excellent performance output of MN-FTENG proposed in this study makes it have significant advantages in capacitor charging or LED driving, which is sufficient for application in various fields. The experiments in this chapter prove that MN-FTENG has the ability to generate electricity and drive electrical appliances by itself. In the future, when developing the HMI system, this characteristic can drive the small electrical appliances in the system to achieve the purpose of battery-free driving.

### 3.4. Force Sensitivity Test

**Figure 9:**
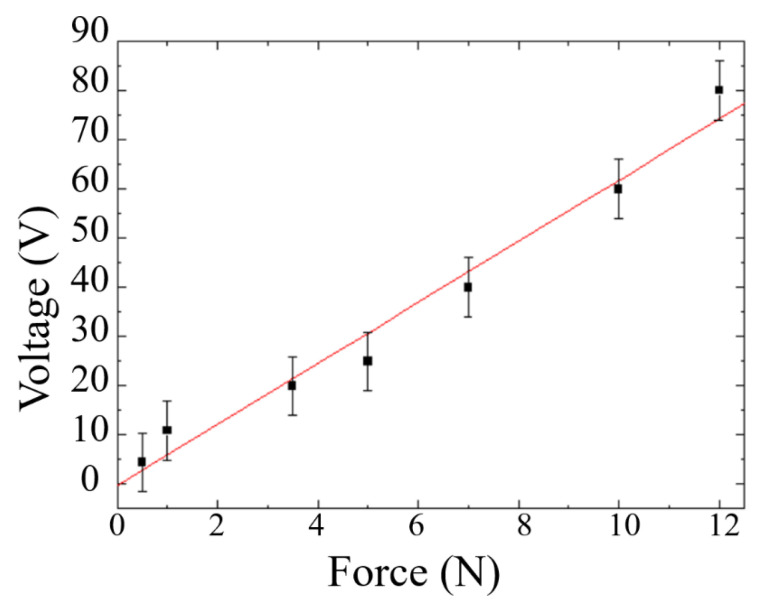
The output voltage of MN-FTENG under various forces, the slope of the sensitivity is 7 V/N, which is significantly higher than other Mechanical energy harvester. Additionally, the coefficient of determination of this experiment is 0.97, which shows high reliability of MN-FTENG’s force sensitivity. The force comes from the weight dropping from a specified height (3 cm, 5 cm, 7 cm, and 10 cm) and is measured by the load cell. The same force acts on MN-FTENG and the output voltage is measured by HIOKI. The measured force range is 0.5 N to 10 N, and the voltage range is 5 V to 80 V.

The MN-FTENG fabricated in this study has quite high mechanical sensitivity due to the deformation and recovery of the PDMS microstructure. As a sensor, it can have different outputs and responses according to the magnitude of the external force. The output voltage of MN-FTENG under various forces is shown in Figure 9. The force range is between 0.5 N to 10 N and the output voltage range is between 5 V to 60 V. It reveals that its output and force have a very high mechanical sensitivity 7 V/N, which is much higher than other mechanical energy harvesters, as listed in Table 2 since FTENG with high deformation can convert more mechanical energy into electric energy. Additionally, the coefficient of determination of R^2^ = 0.97. Based on this calculation, the 16 N will have an output of approximately 80 V, which is in line with the measurement results in Section 3.1. This shows that MN-FTENG can be used as a sensor to measure the force differences by the output signal of MN-FTENG. Higher mechanical sensitivity indicates better sensing ability for tiny motions, which also means that MN-FTENG is more suitable for capturing tiny human motions, such as arm bending and knee bending, compared to other mechanical energy harvesters. Additionally, a coefficient of determination of 0.97 means a sufficiently stable sensing capability.

Based on the output performance in Section 3.1, Section 3.3, Section 3.3 and Section 3.4, MN-FTENG is sufficient to be a mechanical energy harvester for daily use and applied to wearable HMI. In the following sections, we will actually test the benefits of MN-TENG for human motion capture and whether it can be applied to HMI.

### 3.5. Human Motion Detection

**Figure 10:**
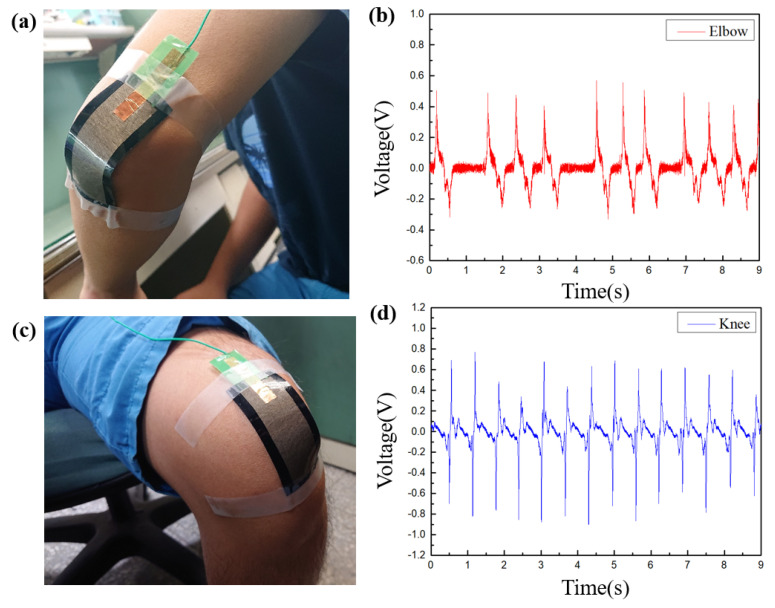
The electric signal of human body motion captured by MN-FTENG, (**a**,**b**)the voltage of the elbow motion signal captured by MN-FTENG reaches 0.6 V; (**c**,**d**) The knee joint reaches 0.8 V. With the result of Figure 9, it also shows the force of squeezing MN-FTENG during elbow bending and knee bending is about 0.05 N and 0.1 N by extrapolation.

In this section, the motion detection of large joints such as elbows and knees will be carried out. As shown in Figure 10a,c, MN-FTENG of 30 mm × 80 mm is used to make patches and fixed to the elbow and knee joints. The highest voltage of the elbow motion captured by MN-FTENG reaches 0.6 V, and its voltage output will change correspondingly with the speed of hand movement, while Figure 10b,d illustrate that there will be slight differences between the waves and the spacing due to the instability of the motion frequency. The motion of the knee joint and its power generation waveform, it is worth noting that the large area of the knee joint also increases the voltage to 0.8 V. With Figure 9, it can be known by extrapolation that the force of squeezing MN-FTENG when the elbow bending is about 0.05 N, and the knee is 0.1 N. Since the elbows and knees have little external resistance when flexed, this output is in line with the force that MN-FTENG should be subjected to.

The height and density of the waveform reflect the force, speed and frequency of human movement. In the future, the user’s force, speed or frequency can be judged through different waveforms, and then the user’s current movement situation can be understood. To sum up, the MN-FTENG developed in this research has good electrical properties, flexible properties and durable structure. It can reliably collect motion waveforms at joints in human motion detection and has the potential to be applied to wearable devices. It can be used as a self-powered sensor for human movement to meet the development of multi-sensor technology in the IoT era, or as an activity detection for post-injury rehabilitation, or to develop a HMI system for human movement control equipment. Next, we will develop the control system of MN-FTENG for wearable HMI.

### 3.6. HMI and Its Applications

**Figure 11:**
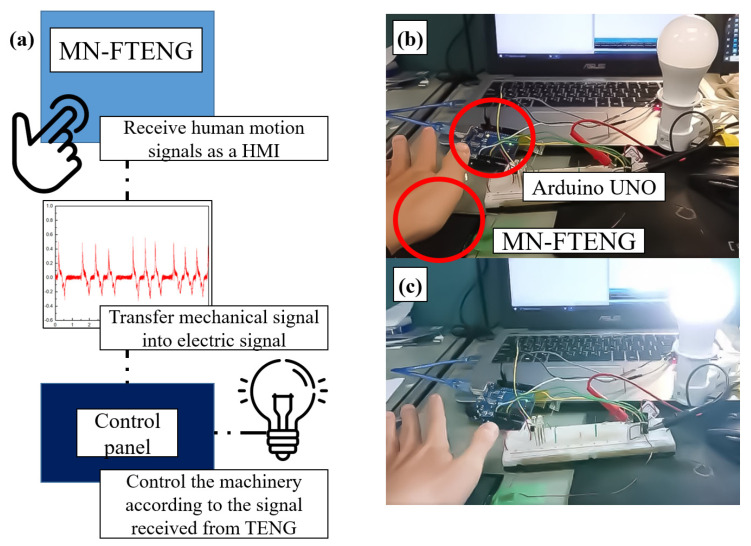
MN-FTENG is used as a self-generating sensor for HMI to drive machinery. (**a**) MN-FTENG converts the motion signal of the human body into electrical signal and inputs it into the control panel, so that it controls the connected device. (**b**) After touching MN-FTENG, MN-TENG will output signal to Arduino UNO, and the Arduino UNO will (**c**) turn on or off the light switch after receiving the signal.

With the feature of MN-FTENG above, through the simple connection between MN-FTENG, control panel and the mechanical device, it is possible to control the lighting of lamps, start the motor, reverse rotation, stop and other commands by clicking, touching of other human motion while MN-FTENG converts the motion signal of the human body into electrical signal and inputs it into the control panel, so that it controls the connected device, as shown in Figure 11a. In this experiment, we combine the MN-FTENG, Arduino UNO and a light bulb as a control system. Whenever the user slightly touches the MN-FTENG, the MN-FTENG will convert the user’s action into an electrical signal and send it to the Arduino UNO to control the light bulb to turn on or off, as shown in Figure 11b,c. Because the mechanical harvester has the characteristics of generating and sensing by itself, it can use this feature to generate signals and provide power for control components. In this system, MN-FTENG does not need to be powered on all the time to operate like most other high-sensitivity HMIs, and can start or drive other devices by relying on the power it captures. This is a very energy efficient way to save energy in systems that require a lot of HMIs.

## 4. Conclusions

In this study, we develop the FTENG as a mechanical energy harvester for HMI sensors. We use a coated with PDMS using commercially available PET and conductive cloth. FTENG with high mechanical sensitivity is used as a self-powered sensor with high flexibility and stretchability, and the microstructure array PMMA mold is fabricated by laser engraving, and the fiber-base triboelectric nanogenerator with microneedle structure (MN-FTENG) is fabricated by demolding. The open-circuit voltage (Voc) and the short-circuit current (Isc) of MN-FTENG with microstructure increase from 54.6 V and 26.16μA to 73.6 V and 36 μA, respectively, which are 34% and 37% higher than FTENG without microneedle structure. Additionally, the output power of MN-FTENG reaches 1.296 mW with 89% higher than that of flat FTENG. In the durability test, the MN-FTENG still maintains a good output performance in 5000 tapping cycles. In the structural cleaning test, good performance stability was also maintained, and the voltage difference was within ±5 V. In the capacitor charging test, MN-FTENG can charge 3.3 μF to 3.3 V within 15 s and MN-FTENG can also light up 90 LEDs. The MN-FTENG has a high and stable mechanical sensitivity of 7 V/N with a coefficient of determination of 0.97 which is higher than most other mechanical harvesters. The above performance is high enough for MN-TENG to be used as a mechanical energy harvester for HMI. As a human body joint motion mechanical energy harvester, MN-FTENG can harvest the motion mechanical energy of the elbow and knee joints as a voltage signal to sense human motion. MN-PET-TENG acts as a self-powered HMI, which can harvest the energy during human motion and store it in capacitors to drive device actions. Additionally, through the control device, the movement of the user’s body is used to operate the machine and turns on or off the LED light through the MN-TENG for potential IoT application.

## Figures and Tables

**Table 1:** Comparison of fiber based TENG.

Material	Voc	Isc	Dimension	Ref.
Silicone-PVA	42.9 V	0.51 μA	1D	[22]
Polyester-Ni-Parylene	50 V	4 μA	2D	[23]
PTFE-Ag	50.6 V	0.29 μA	3D	[24]
PET-PDMS-Al	54.6 V	26.16 μA	2D	This research
PET-MNPDMS-Al	73.6 V	36.00 μA	Enhanced 2D	This research

**Table 2:** Comparison of fiber-based MN-TENG with fiber EMG and PENG.

Mechanical Energy Harvester	Voc	Isc	Force Sensitivity	Material	Ref.
EMG	9 mV	10 mA	0.56 V/Hz	Polyurethane/iron	[32]
PENG	4.0 V	2.6 μA	0.27 V/N	P(VDF-TrFE) micropillar array	[33]
Fiber-TENG	73.6 V	36.0 μA	7 V/N	PET + PDMS/Al	This research

## Data Availability

Data are the coauthors’ research results and drawing.
